# The association between empathy and burnout in medical students: a systematic review and meta-analysis

**DOI:** 10.1186/s12909-024-05625-6

**Published:** 2024-06-07

**Authors:** P. Cairns, A. E. Isham, R. Zachariae

**Affiliations:** 1 Unit for Psycho-Oncology & Health Psychology, Department of Psychology and Behavioural Sciences, Aarhus University, Aarhus, Denmark; 2 Research and Development Department, Division of Mental Health Services, Akershus University Hospital, Sykehusveien 25, 1478 Nordbyhagen, Norway; 3 Department of Oncology, Aarhus University Hospital, Aarhus, Denmark

**Keywords:** Empathy, Burnout, Medical students, Systematic review, Meta-analysis

## Abstract

**Background:**

Burnout levels in medical students are higher than in other student groups. Empathy is an increasingly desired outcome of medical schools. Empathy is negatively associated with burnout in physicians. Our objective was to quantitatively review the available literature on associations between empathy and burnout in medical students, and to explore associations between specific empathy aspects (cognitive and affective) and burnout sub-dimensions (emotional exhaustion, depersonalization and personal accomplishment).

**Methods:**

A comprehensive search of the literature published up until January 2024 was undertaken in the PubMed, EMBASE, CINAHL, The Cochrane Library, and PsycINFO databases. Two independent reviewers screened 498 records and quality-rated and extracted data from eligible studies. The effect size correlations (ESr) were pooled using a random-effects model and between-study variation explored with meta-regression. The review was preregistered with PROSPERO (#CRD42023467670) and reported following the PRISMA guidelines.

**Results:**

Twenty-one studies including a total of 27,129 medical students published between 2010 and 2023 were included. Overall, empathy and burnout were negatively and statistically significantly associated (ESr: -0.15, 95%CI [-0.21; -0.10], *p* < .001). When analyzing sub-dimensions, cognitive empathy was negatively associated with emotional exhaustion (ESr: -0.10, 95%CI [-0.17; -0.03], *p* = .006) and depersonalization (ESr: -0.15, 95%CI [-0.24; 0.05], *p* = .003), and positively associated with personal accomplishment (ESr: 0.21, 95%CI [0.12; 0.30], *p* < .001). Affective empathy was not statistically significantly associated with emotional exhaustion, depersonalization or personal accomplishment. Supplementary Bayesian analysis indicated the strongest evidence for the positive association between cognitive empathy and personal accomplishment. Response rate and gender moderated the relationship so that higher response rates and more male respondents strengthen the negative association between empathy and burnout.

**Conclusion:**

Greater empathy, in particular cognitive empathy, is associated with lower burnout levels in medical students. This appears to be primarily driven by cognitive empathy's positive association with personal accomplishment.

**Protocol registration:**

#CRD42023467670

**Supplementary Information:**

The online version contains supplementary material available at 10.1186/s12909-024-05625-6.

## Background

The purpose of this systematic review and meta-analysis was to examine the relationship between empathy and burnout in medical students. Burnout, defined as a state of emotional exhaustion, depersonalization, and a reduced sense of personal accomplishment [1], has become a pervasive issue within the medical field. One systematic review and meta-analysis of 4,664 international medical residents reported an overall burnout prevalence rate of 35.7% [2]. In US physicians, reports indicate a prevalence of burnout of 37.9%, compared to 27.8% in the general population [3]. Research indicates that burnout prevalence rates in medical students range from 7 to 75.2%, depending on the country in which the study was carried out, the instruments used and the cutoff-criteria for burnout symptomatology [4], with an overall suggested prevalence rate of 37.23% [5]. One US study found that 49.6% of medical students may experience burnout, compared to 35.7% of U.S. college graduates aged 22 to 32 [6]. Prospective studies suggest that burnout may increase from the first year of medical school to the final year [7, 8].

Burnout among medical students has been found to be associated with poorer academic performance, increased rates of substance abuse, and impaired mental health, which could impact future physicians' ability to provide high quality, compassionate patient care [9]. Individual studies suggest that burnout may also be negatively associated with medical student empathy [10]*.*

Although multiple definitions of empathy have been suggested [11], it is generally considered to have three dimensions: cognitive empathy in which physicians use their cognitive abilities to take the perspective of their patient, an affective component in which physicians feel the emotions they believe their patient is experiencing, and a behavioral component in which the physicians communicate their understanding [12]. A consensus has grown around the definition of therapeutic empathy in recent years, defined as '*a physician's ability to understand the patient, communicate that understanding and act upon it in a therapeutic way.*' [13]. This definition prioritizes the cognitive understanding of the patient over the affective feeling of their emotions [14].

Medical students are increasingly expected to use and develop empathy as part of their medical education, as shown in curriculums specifically highlighting communication skills and empathy [15, 16]. This is important given that empathetic healthcare consultations are associated with increased physician-patient trust [17], improved psychological and physical patient outcomes [18, 19], and an increase in patient satisfaction by lowering patient anxiety and distress [19, 20].

Therapeutic empathy may have benefits for physicians as well as patients. One systematic review of healthcare workers found negative associations between empathy and subdimensions of burnout: emotional exhaustion, depersonalization and reduced personal accomplishment [21]. Specifically, the authors found a negative association between perspective-taking (cognitive empathy) and depersonalization, and a positive association between perspective-taking and personal accomplishment. Similarly, they found a negative association between empathic concern (affective empathy) and depersonalization and a positive association between empathic concern and personal accomplishment. Emotional exhaustion was not related to either perspective-taking or empathic concern. Potential moderators were not explored in this systematic review, but a number of individual studies have suggested that gender [22] and age [23], amongst other variables, are also associated with empathy and burnout with the possibility of acting as moderators. Empathic concern (affective empathy) is higher in women compared to men, and perspective-taking (cognitive empathy) is higher in women and younger people compared to men and older people [23].

Despite having similarly high burnout levels to physicians, no systematic review and meta-analysis has explored the possible association between empathy and burnout in medical students. Given the high levels of burnout in medical students and an increasing focus on empathic skills in medical education, the aim of this systematic review and meta-analysis was to explore the possible association between empathy and burnout in medical students. This knowledge could be important for developing preventive strategies to avoid burnout, maintaining the mental health of medical students, promoting medical career sustainability and ensuring quality of care for present and future patients.

### Purpose of the study

This systematic review and meta-analysis aimed to provide a quantitative synthesis of the existing literature on the relationship between empathy and burnout in medical students. We addressed the following research questions:

- What is the overall association between empathy and burnout in medical students?
- Are specific dimensions of empathy (cognitive, emotional, behavioral) differentially related to specific burnout dimensions (emotional exhaustion, depersonalization, reduced personal accomplishment)?
- What moderating factors may influence the relationship between empathy and burnout in medical students (e.g. gender, age, study level, region)?

## Methods

The protocol for the present study was preregistered in the International Prospective Register of Systematic Reviews (PROSPERO) (#CRD42023467670) [24]. The present study deviates from the protocol in the following ways: a) due to an existing systematic review on healthcare workers, physicians were no longer included as a population of interest, and b) due to a lack of studies, compassion fatigue was not included as a focus of this review. The review was conducted in accordance with the guidelines for Meta-Analysis Of Observational Studies in Epidemiology (MOOSE) [25] and is reported in line with the Preferred Reporting Items for Systematic Reviews and Meta-Analyses (PRISMA) guidelines [26].

### Search strategy and selection criteria

A comprehensive literature search was conducted on September 22, 2023 as per protocol, with an updated search conducted on January 10, 2024. The updated search only included medical students because physicians were no longer a population of interest. No publication date restrictions were applied. The electronic databases searched were: PubMed, EMBASE, CINAHL, The Cochrane Library, and PsycINFO. Where possible, relevant MeSH (Medical Subject Headings) terms or MeSH term equivalents were included in each database search. The specific search terms were: medical student* AND empathy AND burnout.

The study inclusion criteria were guided by the PICO (Population, Intervention/Exposure, Comparator, Outcome) approach [27]. Population: Medical students; Exposure: Medical school; Comparator: N/A; Outcome: Empathy and burnout assessed with a standardized, validated measurement scale. The study exclusion criteria were Population: Non-medical students; Exposure: Non-medical school; Comparator: N/A; Outcome: Non-empathy and burnout measures. Empathy and burnout assessed with non-standardized, unvalidated measurement scales.

We included correlational studies, including cross-sectional and longitudinal, prospective survey-based studies. Furthermore, only English-language papers published in peer-reviewed journals were considered eligible. We chose not to include non-English papers, as this might introduce biases related to language, publication bias, methodological heterogeneity, and challenges in access and quality assessment. While inclusivity is important, the potential for bias introduced by non-English papers outweighs the benefits of attempting a more comprehensive review. We excluded randomized controlled trials (RCTs), qualitative studies, case studies, open trials, uncontrolled trials, reviews and study protocols. The reason for this was that they were not designed to collect correlational data and test correlational hypotheses, or that they do not provide quantitative data. Additionally, including data from trials, e.g., baseline or control group data, may provide less generalizable data due to often highly selected study samples and that data are likely to be influenced by the experimental setup of such trials. Grey literature, for example, conference abstracts, trial registrations, dissertations and studies with N<10 was also not considered eligible.

The literature search and data extraction were conducted using the Covidence systematic review software [28]. In the first round of screening, PC and AEI independently screened the title and abstract of all identified references and excluded ineligible studies. In the second round of screening, the full text of the remaining studies were evaluated independently by PC and AEI and reasons for exclusion were registered. After each screening, the two authors discussed discrepancies, and reached a negotiated decision. Uncertainties and disagreements were discussed with the last author (RZ).

### Quality assessment

A methodological quality assessment was undertaken independently by two authors (PC and AEI) for all included studies, using the National Institutes of Health Quality Assessment Tool for Observational Cohort and Cross-Sectional Studies [29]. The quality terms included whether the studies had a clearly defined research question and study population, whether the participation rate of eligible participants was at least 50%, whether the subjects were recruited from the same or similar populations, whether inclusion and exclusion criteria were used for all participants, whether a sample size justification such as a power description was provided, and if key confounding variables were measured and adjusted for statistically, among other questions.

### Data extraction

Data extraction was performed independently by two authors (PC and AEI) and included authors, publication year, empathy aspect (cognitive, affective or behavioral), burnout dimension (emotional exhaustion, depersonalization or personal accomplishment), correlation statistic (Pearson's *r*, Spearman's *ρ*, or standardized *β* values), sample size, effect direction, whether the correlation was adjusted for other covariates (yes or no), number of covariates, study design (cross-sectional or longitudinal), response rate (as percentage), whether the relationship between empathy and burnout in medical students was the primary focus of the study (yes or no), sampling method (convenience, random), sample mean age, gender of participants (percent women), country, region (e.g., North America, Middle East, Europe, Asia), study level (early, late, or mixed), empathy scale, empathy subscale, burnout scale and burnout subscale. A meta-analysis was conducted when a minimum of three studies assessing an association between an empathy and a burnout dimension were available.

### Categorization of empathy and burnout data

Empathy in the context of medical education is a multidimensional construct that encompasses cognitive, emotional, and behavioral components [12]. Cognitive empathy refers to the ability to understand the thoughts and perspectives of others, emotional empathy involves feeling and sharing the emotions of others, and behavioral empathy entails demonstrating empathetic behaviors, such as active listening and providing emotional support [30]. In the caring professions, 11 empathy measurement tools are available [31]. In order to make a meta-analysis possible and reduce the complexity of the findings, all empathy questionnaires were categorized as measuring either cognitive, affective or behavioral empathy. The study characteristics table (Table 1) provides the measurement tool used in each study and the empathy aspect categorized by the authors.

**Table 1:** Characteristics of the included studies

**Study**	**N**	**Study level**	**% women**	**Empathy** **Aspect**	**Burnout dimensions**	**Empathy instrument**	**Burnout instrument**	**Primary focus of study**	**Response rate**	**Country**
Bigdeli et al. 2021 [32]	167	NR	58	Cognitive	Global	JSE-S	MBI-GS	Yes	NR	Iran
Bohler et al. 2021 [33]	80	Late	63.7	Cognitive, Affective	EE, DP, PA	EQ	MBI-GS	No	NR	Australia
Brazeau et al. 2010 [34]	124	Late	48	Cognitive	EE, DP, PA	JSE-S	MBI-HSS	Yes	71.8	USA
Cangussu Silva et al. 2018 [35]	776	Mixed	54.4	Global	EE, DP, PA	EI, ESWIM	OLBI	No	76.9	Brazil
Capdevila-Gaudens et al. 2021 [36]	5216	Mixed	76.3	Cognitive	EE, DP, PA	JSE-S	MBI-S	No	12.42	Spain
Carrard et al. 2022 [37]	886	Mixed	68.4	Cognitive, Affective, Behavioral	EE, DP, PA	JSE-S, QCAE, AMSP	MBI-S	Yes	49.41	Switzerland
Chae et al. 2017 [38]	127	Early	47.24	Cognitive	EE, DP, PA	JSE-S	MBI-S	Yes	74.27	South Korea
Damiano et al. 2017 [39]	106	Early	54.7	Global	Global	ESWIM	OLBI	Yes	37	USA
DeWitt et al. 2016 [40]	660	Early	54.5	Affective	Global	IRI	CBI	No	31	Australia
Dyrbye et al. 2021 [41]	14126	Late	52	Global	EE, DP	IRI	OLBI	Yes	NR	USA
Gradiski et al. 2022 [42]	171	Mixed	53.2	Cognitive	EE, DP, PA	JSE-S	MBI-GS	Yes	77	Croatia
Greenmyer et al. 2022 [43]	162	Early	53	Affective	EE, DP	TEQ	OLBI	No	52.3	USA
Kilic et al. 2021 [44]	342	Mixed	73.98	Cognitive, Affective	EE, DP, PA	IRI	MBI-S	Yes	25	Belgium
Lucchetti et al. 2018 [45]	138	Early	52.1	Global	EE, DP	ESWIM	OLBI	No	48.6	USA
Paro et al. 2014 [10]	1350	Mixed	52.9	Cognitive, Affective	EE, DP, PA	IRI	MBI-HSS	Yes	81.8	Brazil
Shin et al. 2022 [46]	1293	Mixed	44.7	Cognitive, Affective	EE, DP, PA	IRI, JSE-S	MBI-S	Yes	NR	South Korea
Stosic et al. 2022 [47]	76	Early	59	Cognitive	Global	TAPPA	ProQOL	Yes	13	USA
Suh et al. 2019 [22]	271	Mixed	27.3	Cognitive	EE, DP, PA	JSE-S	MBI-GS	Yes	95	South Korea
von Harscher et al. 2018 [48]	353	Early	45	Affective	EE, DP, PA	IRI	MBI-HSS	Yes	NR	USA
Wercelens et al. 2023 [49]	150	Late	70.7	Cognitive, Affective	EE, DP, PA	IRI	MBI-HSS	Yes	54	Brazil
Wu et al. 2022 [50]	588	Mixed	62	Cognitive, Affective	EE, DP, PA	BES	LBS	Yes	98	China

*Abbreviations*: *NR* Not Reported, *EE* Emotional Exhaustion, *DP* Depersonalization, *PA* Personal Accomplishment, *JSE-S* Jefferson Scale of Empathy – Student, *EQ* Empathy Quotient, *EI* Empathy Inventory, *ESWIM* Empathy, Spirituality, and Wellness in Medicine Scale, *QCAE* Questionnaire of Cognitive and Affective Empathy, *AMSP* Ability to Modify Self-Presentation Scale, *IRI* Interpersonal Reactivity Index, *TAPPA* Test of Accurate Perception of Patients’ Affect, *BES* Basic Empathy Scale, *EC* Empathic Concern, *PT* Perspective Taking, *MBI-GS* Maslach Burnout Inventory - General Survey, *MBI-HSS*, Maslach Burnout Inventory - Human Services Survey, *OLBI* Oldenburg Burnout Inventory, *MBI-S* Maslach Burnout Inventory – Student, *CBI* Copenhagen Burnout Inventory, *SMBM* Shirom-Melamed Burnout Measure, *ProQOL* Professional Quality of Life, *LBS* Learning Burnout Scale

Burnout among medical students is usually conceptualized within the framework of the Maslach Burnout Inventory (MBI), which identifies three key dimensions: emotional exhaustion, depersonalization, and reduced personal accomplishment [1]. Emotional exhaustion refers to feelings of fatigue and emotional depletion, depersonalization involves cynicism and detachment from patients, and reduced personal accomplishment reflects a diminished sense of personal achievement and competence [1]. However, at least four other measurement tools for occupational burnout exist [51] with various dimensions, broadly aligning with the three dimensions of the MBI. All burnout questionnaires were categorized as measuring either emotional exhaustion, depersonalization or personal accomplishment. The study characteristics table (Table 1) provides the measurement tool used in each study and the burnout dimensions it was categorized as covering.

The three dimensions of burnout have different directions. For emotional exhaustion and depersonalization, greater scores signify greater burnout. For personal accomplishment, higher scores indicate less burnout. Therefore, when examining the association between global empathy and global burnout, correlations between empathy and personal accomplishment were reverse scored. When calculating the associations between the specific aspects of empathy and the various dimensions of burnout, personal accomplishment was not reverse scored.

### Meta-analytic strategy

The effect size correlation (ESr) was used as the standardized effect size for the association between empathy and burnout. If correlations were not reported directly, ESr was converted from other data, for example, differences between means and standard deviations, regression coefficients, numbers or rates of study participants in relevant groups, χ^2^, *F*, or *t* statistics using various formulas. The calculations were conducted independently by two authors (PC and AEI) and checked by a third author (RZ) in case of disagreement. In case of missing data from the published report, the data was requested from the authors.

Effect sizes were calculated for both the unadjusted bivariate associations and the associations found in multivariate analyses adjusted for other covariates. Correlations between global empathy and global burnout were operationalized as the averaged correlations across the various individual dimensions for each study. The ESr was also used as a standardized effect size for the association between the different aspects of empathy (cognitive, affective and behavioural) and the dimensions of burnout (emotional exhaustion, depersonalization and personal accomplishment). The pooled effect size (ESr) and its 95% confidence interval was calculated using a random effects model. Heterogeneity was explored by calculating the *I*^*2*^ statistic. The *I*^2^ statistic is an estimate of the variance in a pooled ES that is accounted for by heterogeneity, i.e., true differences between effect sizes rather than sampling error [52]. We also calculated Tau (*T*), which represents the standard deviation of the true effect sizes, and the 95% prediction interval. The prediction interval takes both the random error and the systematic variance into consideration and quantifies the distribution of the ESs, indicating the range that 95% of results of future studies (from the same family of studies) are expected to fall within [53].

Publication bias, i.e., the tendency for statistically significant findings in the hypothesized direction to be more likely to be published, is a widespread problem in meta-analyses [54]. Although publication bias may be less likely in correlational than effect studies, we explored the possibility of using funnel plots and Egger's tests [55], but only when K > 10 (K = number of studies) [54]. If the results were suggestive of possible publication bias, we planned a sensitivity analysis adjusting the effect sizes using the Duval and Tweedie trim-and-fill method [56].

Possible sources of heterogeneity were explored with moderator analyses. When data were available for 10 independent samples or more, the possible influence of both continuous moderators (i.e., mean sample age, percent of women in the sample, response rate) and dichotomous moderators (i.e., student study level (late vs. early) and study quality (good or fair vs. poor)) were analyzed with meta-regression (computational model: maximum likelihood). For dichotomous moderators, the variable had to show sufficient variability, i.e. K > 3 in the smallest category. The R^2^ equivalent was calculated for moderators reaching statistical significance.

All analyses were conducted using Comprehensive Meta-Analysis v4 [57] and various formulas in Microsoft Excel.

### Supplementary Bayesian analyses

To aid the interpretation of the results, a Bayesian Model-Averaged meta-analysis [58] was conducted. The procedure examines the results of four models: a) Fixed-effect null hypothesis (fH_0_), b) fixed-effect alternative hypothesis (fH_1_), c) random-effects null hypothesis (rH_0_), and d) random effects alternative hypothesis (rH_1_). Bayesian Model-Averaged analysis thus avoids selecting either a fixed- or random-effects model and addresses two questions considering the observed data: What is the plausibility that the overall effect is non-zero and the ES are heterogeneous? An uninformed prior probability was chosen, i.e., 25%, of each of the four models, and 2000 iterations were used. With regard to parameter distributions, previously recommended defaults were chosen [58]. Thus, a zero-centered Cauchy prior with a scale of 0.707 for the ES was used. For the between-study variation, an empirically informed prior distribution on non-zero between-study deviation estimates based on standardized mean difference ESs from 705 meta-analyses published in Psychological Bulletin between 1990 and 2013 was used [59]. This distribution has been approximated by an Inverse-Gamma (1, 0.15) prior on the standard deviation (Tau) [58]. For each analysis, we calculated the Bayes Factor (BF) [60], which represents the posterior probability of the alternative hypothesis (H_1_) relative to the probability of the null hypothesis. Based on the BF, the strength of the evidence was then categorized as "weak", “moderate”, “strong”, “very strong”, and “decisive” [61]. The Bayesian analyses were conducted with the computer software JASP (Version 0.17.1) [62].

## Results

### Search results

**Fig. 1:**
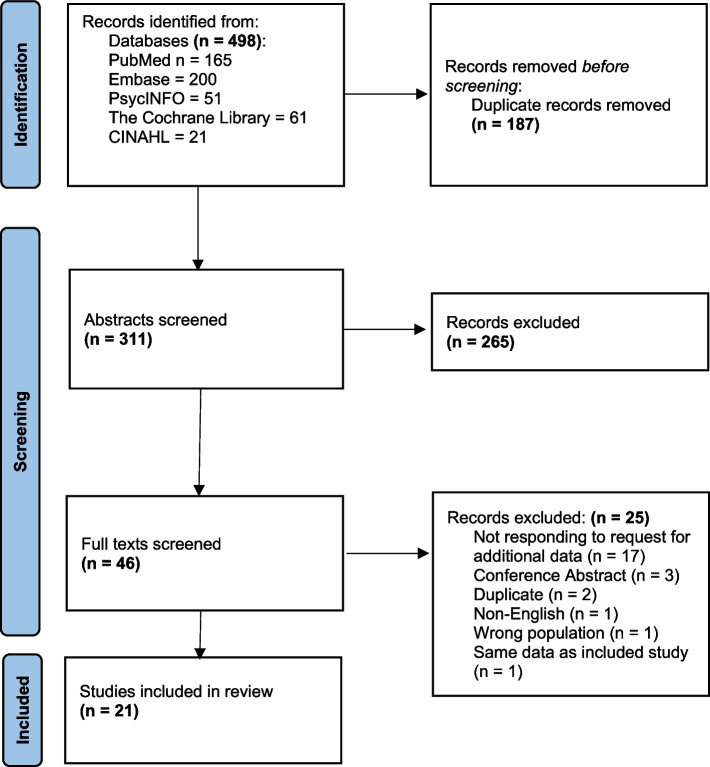
Preferred Reporting Items for Systematic Reviews and Meta-Analyses diagram

A total of 498 articles were identified through digital database searches. After removal of duplicates, a total of 311 records were eligible for title and abstract screening. A total of 265 records were excluded after screening, leaving 46 articles eligible for full text screening. 25 articles were then excluded primarily due to “not responding to request for additional data” (68%), leaving 21 papers that were included in the systematic review. Ninety percent agreement was achieved by reviewers during the abstract review process and 95% agreement during full-text screening. All disagreements were resolved through negotiation. This negotiation involved the two reviewers PC and AEI providing the reason for their decision to include or exclude the relevant study. These reasons were then double-checked by both PC and AEI reviewing the individual study collaboratively. This led to agreement on whether to include or exclude the study in question. The study selection process is visualized in Fig. 1. Authors of 25 studies were contacted and asked to provide additional data. Seven authors replied and provided the requested data.

### Study characteristics

The participant characteristics, the empathy and burnout dimensions examined, the measurement tools used, and other characteristics of the included studies are summarized in Table 1. The identified studies reported on 21 independent samples including a total of 27,129 medical students, with sample sizes ranging from 76 [47] to 14,126 [41]. The included articles were published between 2010 and 2023. The studies were broadly geographically distributed, with 7 studies conducted in North America, 4 in Asia, 4 in Europe, 3 in South America, 2 in Oceania, and 1 in the Middle East. Mean sample ages ranged from 19.9 [50] to 27.7 [41] years, with an overall weighted mean sample age of 25.2 years. The majority (90.4%) of the studies (*K* = 19) used a cross-sectional design, and 9.6% (*K* = 2) used a longitudinal design. As the majority of studies employed cross-sectional surveys across multiple study years, it was not possible to construct a continuous study year variable. We, therefore, categorized the study year as either early (years 1-3), late (years 4-6+), or mixed (1-6+).

### Quality rating

See supplementary table S1 for an overview of the quality ratings of each study. Two studies were assessed to be of good quality (>9 criteria met), and 19 studies to be of fair quality (5 to 9 criteria met). Studies received high ratings when they presented a clear definition of the research question and study population, when there was a sufficient timeframe between longitudinal measurements, and when a sample size justification, i.e., statistical power calculation, was reported.

### Overall association between empathy and burnout

The most commonly used scale to assess empathy was the Jefferson Scale of Empathy - Student version (K = 8), followed by the Interpersonal Reactivity Index (K = 7). The most commonly used scale to assess burnout was the Maslach Burnout Inventory – Student version (K = 5) and the Oldenburg Burnout Inventory (K = 5), followed by the Maslach Burnout Inventory – General Survey (K = 4) and the Maslach Burnout Inventory – Human Services Scale (K = 4). As mentioned above, empathy was characterized as having cognitive, affective, and behavioral components. Due to a lack of studies (K = 1), the behavioral aspect of empathy was not explored in this meta-analysis.

**Fig. 2:**
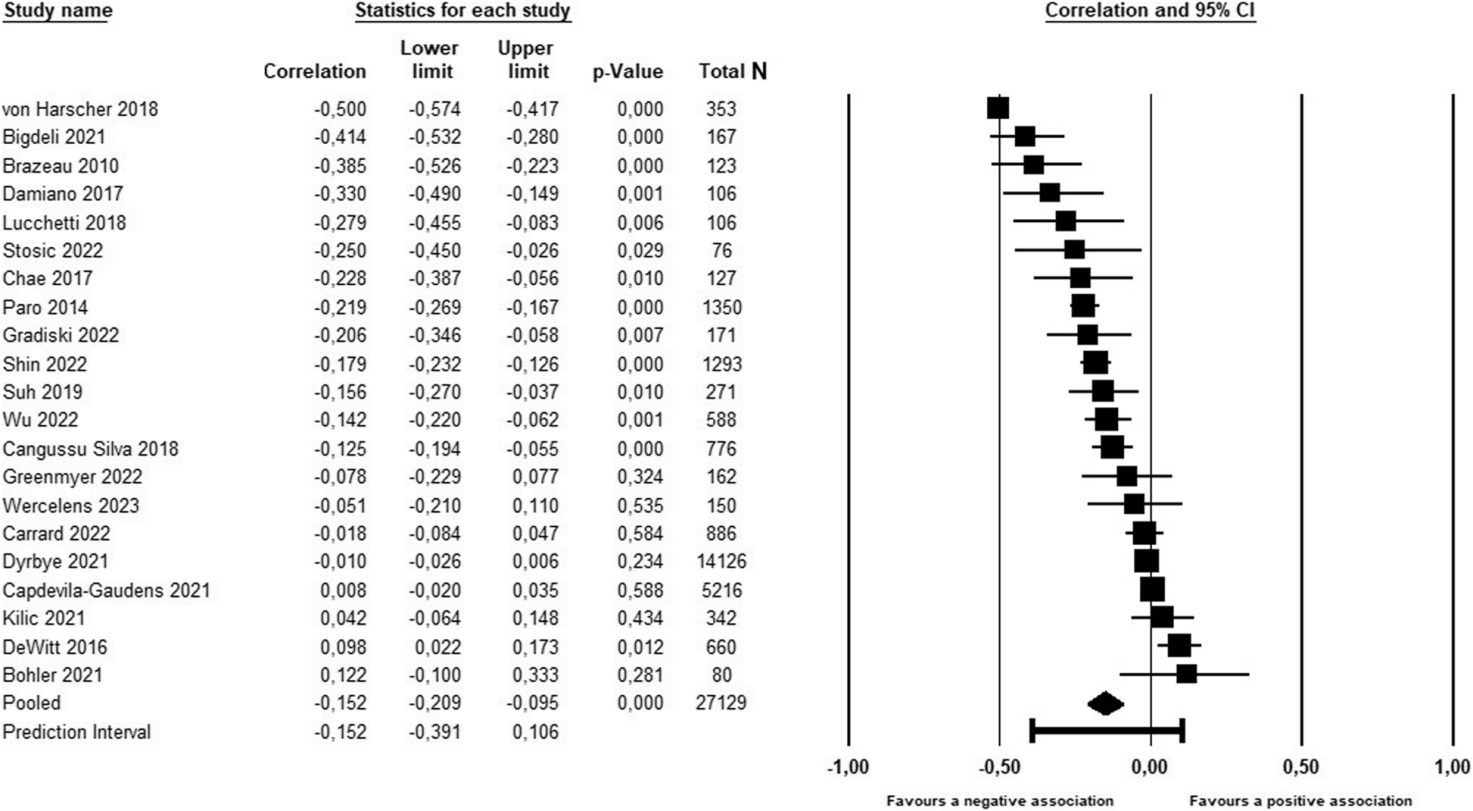
Forest plot of the association between empathy global and burnout global

As shown in Table 2 and Fig. 2, global empathy was negatively associated with global burnout in medical students, with the pooled correlation corresponding to a small effect size [63]. The supplementary Bayesian analysis indicated that, based on the available evidence, the alternative hypothesis, i.e., that the association between global empathy and global burnout is non-zero, was approximately 35 times more likely than the null hypothesis, corresponding to “very strong evidence” [61].

**Table 2:** Associations between empathy and burnout symptoms

**Empathy parameter**	**Burnout parameter**	**K**	**N**	***I***^**2**^	***T***	**ESr**	**95%CI**	***p***	**95%PI**	**BF**	**Evidence**
Global	Global	21	27129	93.1	0.14	-0.15	-0.21; -0.10	**<0.001**	-0.39; 0.11	35.1	✶✶✶✶
-	Emotional exhaustion	17	26121	91.9	0.10	-0.08	-0.14; -0.03	**0.004**	-0.10; 0.14	0.3	◯◯
-	Depersonalization	17	26120	95.4	0.14	-0.19	-0.25; -0.12	**<0.001**	-0.46; 0.12	39.0	✶✶✶✶
-	Personal accomplishment	13	10950	93.9	0.15	0.20	0.11; 0.28	**<0.001**	-0.15; 0.50	86.9	✶✶✶✶
Cognitive	Emotional exhaustion	12	10598	89.6	0.11	-0.10	-0.17; -0.03	**0.006**	-0.35; 0.16	0.6	◯
-	Depersonalization	12	10597	94.7	0.16	-0.15	-0.24; 0.05	**0.003**	-0.48; 0.22	1.7	✶
-	Personal accomplishment	12	10597	93.8	0.15	0.21	0.12; 0.30	**<0.001**	-0.13; 0.51	310.6	✶✶✶✶✶
Affective	Emotional exhaustion	9	5204	95.9	0.21	-0.02	-0.17; 0.12	0.741	-0.50; 0.46	0.1	◯◯◯
-	Depersonalization	9	5204	95.9	0.21	-0.11	-0.24; 0.04	0.145	-0.56; 0.40	0.2	◯◯
-	Personal accomplishment	8	5042	95.5	0.19	0.10	-0.04; 0.23	0.156	-0.38; 0.54	0.4	◯

Empathy parameter: Global = combination of cognitive empathy, affective empathy, and scales measuring both; Burnout parameter: Global = combination of emotional exhaustion, depersonalization and reverse-scored personal accomplishment; K = Number of independent samples analyzed; N = total number of participants in the analysis; *I*^*2*^ = heterogeneity statistic, i.e., the percent of the variance attributable to systematic (non-random) between-study differences in the effect size; *T* = Tau, i.e., the estimated standard deviation of the true effect size; ESr = effect size correlation; 95%CI = 95% confidence interval of ESR; *p* = *p*-value (two-tailed); 95%PI = 95% prediction interval, i.e., the interval in which 95% of future observations from the same family of studies will fall; BF (the Bayes Factor) [60] represents the posterior probability of the alternative hypothesis (H_1_) relative to the probability of the null hypothesis; Values < 1 indicate evidence in favor of the null hypotheses: ◯ weak (0.33-1.00), ◯◯ moderate (0.10-0.33), ◯◯◯ strong (0.03-0.10), ◯◯◯◯ very strong (0.01-0.03), ◯◯◯◯◯ decisive (< 0.01). Values > 1 correspond to evidence in favor of the alternative hypothesis: ✶ weak (BF=1-3), ✶✶ moderate (3-10), ✶✶✶ strong (10-30), ✶✶✶✶ very strong (30-100), ✶✶✶✶✶ decisive evidence (>100) [61]

As seen in Table 2, the associations between global empathy and the three subcomponents of burnout all reached statistical significance. Again, the correlations were of small magnitude (ESr -0.19 to 0.20) and in the expected directions, with negative associations between global empathy and emotional exhaustion and depersonalization, and a positive association between global empathy and personal accomplishment. While there was “very strong evidence” for depersonalization and personal accomplishment, the results of the Bayesian analysis favored the null hypothesis for emotional exhaustion, albeit only with moderate level of evidence.

### Associations between sub-dimensions of empathy and burnout

As shown in Table 2, the associations between cognitive empathy and the three sub-dimensions of burnout all reached statistical significance. Again, the correlations were of small magnitude (ESr -0.10 to 0.21) and in the expected directions, with negative associations between cognitive empathy and emotional exhaustion and depersonalization, and a positive association between cognitive empathy and personal accomplishment. While there was “decisive evidence” for personal accomplishment and “weak evidence” for depersonalization, the results of the Bayesian analysis favored the null hypothesis for emotional exhaustion, albeit only with weak level of evidence.

As seen in Table 2, the associations between affective empathy and the three sub-dimensions of burnout did not reach statistical significance. The results of the Bayesian analysis favored the null hypothesis with “strong evidence” for emotional exhaustion, “moderate evidence” for depersonalization, and “weak evidence” for personal accomplishment.

### Publication bias

When examining the overall results, i.e., the association between global empathy and global burnout, we found no clear indications of possible publication bias. When inspecting the funnel plot (See supplementary materials, Figure S1), it did not appear particularly skewed, and neither Egger’s regression test (*p* = 0.311) nor the rank correlation tests for Funnel plot asymmetry (*p* = 0.740) reached statistical significance.

### Heterogeneity

As shown in Table 2, the results were characterized by considerable heterogeneity, with *I*^2^ values ranging from 91.9% to 95.9%. This suggests that very high proportions of the variation in the correlations between empathy and burnout are explained by systematic, i.e., non-random, between-study differences. Based on the variation of the true values, the prediction intervals, i.e., the range of values that the results of 95% of future similar studies are expected to fall within, were wide for most association estimates.

### Moderating variables

As seen in Table 3, when exploring the potential sources of heterogeneity, the percentage of women in the sample and the response rate were the most consistent statistically significant moderators of the associations between empathy and burnout, explaining between 25% and 73% of the variation. The positive slopes found for the percentage of women and the associations between global empathy and global burnout and depersonalization, and the negative slope found for the association between global empathy and personal accomplishment, indicates that stronger negative associations between empathy and burnout were found in samples with fewer women, i.e., more men. The slopes found for response rates indicated that stronger associations in the expected direction between global empathy and two of the burnout dimensions were found in studies with higher response rates. The results for the remaining moderators, i.e., sample mean age, student study level (early vs. late), and study quality (good or fair vs. poor) did either not reach statistical significance or could not be analyzed due to insufficient data.

**Table 3:** Moderators of the associations between empathy and burnout (meta-regression)

**Empathy parameter**	**Burnout parameter**	**Moderator**	**K**	**Slope**	**95%CI**	***p***	**R**^**2**^
Global	Global	Sample age	13	-0.00	-0.03; 0.03	0.831	0.00
		Percentage of women	20	0.01	0.00; 0.01	**0.019**	0.25
		Late study level (ref. early level)	11	0.14	-0.10; 0.39	0.251	0.11
		Response rate	17	-0.00	-0.01; 0.00	**0.006**	0.40
		Good study quality (ref. fair)	*Insufficient data*				
Global	Emotional exhaustion	Sample age	10	0.01	-0.01; 0.04	0.226	0.19
		Percentage of women	16	0.00	-0.00; 0.01	0.453	0.04
		Late study level (ref. early level)	*Insufficient data*				
		Response rate	14	-0.00	-0.01; 0.00	0.131	0.16
		Good study quality (ref. fair)	*Insufficient data*				
Global	Depersonalization	Sample age	10	0.00	-0.04; 0.04	0.922	0.00
		Percentage of women	16	0.01	0.01; 0.02	**<0.001**	0.60
		Late study level (ref. early level)	*Insufficient data*				
		Response rate	14	-0.01	-0.01; 0.00	**<0.001**	0.55
		Good study quality (ref. fair)	*Insufficient data*				
Global	Personal accomplishment	Sample age	*Insufficient data*				
		Percentage of women	13	-0.01	-0.01; -0.00	**<0.001**	0.73
		Late study level (ref. early level)	*Insufficient data*				
		Response rate	11	0.00	0.00; 0.01	**0.007**	0.47
		Good study quality (ref. fair)	*Insufficient data*				

Moderator = study and sample characteristics analyzed as possible moderators of the association (ESr) between empathy and burnout, computational model: Maximum likelihood. Criteria: The moderator analyses were conducted with meta-regression when a) the adjusted predictor reached statistical significance (*p* < 0.05), b) when data from ≥ 10 independent samples was available, and, c) in case of dichotomous moderators, when the moderator showed sufficient variability; K = number of independent samples; *Insufficient data*: K < 10 or insufficient variation of dichotomized data (K < 3 in one category)

## Discussion

The purpose of this systematic review and meta-analysis was to examine the relationship between empathy and burnout in medical students. We found a negative, statistically significant association between empathy and burnout in medical students, with a small effect size. This relationship appeared to be primarily driven by cognitive empathy, which was negatively associated with emotional exhaustion and depersonalization and positively associated with personal accomplishment. Affective empathy was not statistically significant with any of the burnout sub-dimensions. Gender moderated the relationship between empathy and burnout such that the negative relationship between empathy and burnout was stronger in samples with more men. Finally, the response rates of included studies also moderated the relationship between empathy and burnout such that stronger, negative association between empathy and depersonalization and a stronger, positive association between empathy and personal accomplishment were found in studies with higher response rates. Sample mean age, student study level, and study quality either did not reach statistical significance or could not be analyzed due to insufficient data.

These results have some shared findings with that of a systematic review and meta-analysis examining empathy and burnout in healthcare workers, specifically doctors and nurses [21]. In this population, shared findings with our results included a negative association between perspective taking (cognitive empathy) and depersonalization, and a positive association with personal accomplishment. Furthermore, they also found no association between empathic concern (affective empathy) and emotional exhaustion. Some results from this population differed to ours. They did not find a significant association between perspective taking (cognitive empathy) and emotional exhaustion, which we did albeit with a Bayes Factor below 1 indicating weak support for the null hypothesis (no association between cognitive empathy and emotional exhaustion). Furthermore, empathic concern (affective empathy) was significantly negatively associated with depersonalization and significantly positively associated with personal accomplishment in their sample, whereas our sample showed no association between affective empathy and any burnout subscale.

The lack of associations or small effect size correlations between emotional exhaustion and cognitive and affective empathy which both we and the review including doctors and nurses [21] present, suggest that emotional exhaustion may not be influenced so much by empathy-related factors, but perhaps things such as high workloads and lack of sleep [64]. Possible explanations for differences between our findings could be that there are differences between how medical students’ and healthcare workers’ affective empathy and burnout interact, or that our use of multiple affective empathy scales compared to their use of the IRI only impacted the results.

Given that empathy involves other-orientated processes, including considering the other person’s perspective and feeling the emotions that the other may be experiencing, the negative association between empathy and depersonalization is less surprising.

Given that cognitive empathy involves taking the other person’s perspective, one could have expected that the main driver of the negative association between cognitive empathy and burnout was a negative association with depersonalization (viewing people as objects rather than human beings). Although this negative association was statistically significant, the main driver of cognitive empathy’s negative association with burnout was clearly a positive association with personal accomplishment. One explanation for this could be that by using cognitive empathy, medical students may be able to give the patients they encounter more personalized care plans that suit the patient’s life situation and values, as well as creating rapport and a sense of trust [65]. Medical students may perceive this as clinical competence, especially if they pass communication-based assessments such as OSCEs [66] as a result of this, and feel a sense of personal accomplishment. This greater sense of personal accomplishment may contribute to feeling less burnt out.

The finding that gender moderated the relationship between empathy and burnout such that the negative relationship between empathy and burnout was stronger in samples with more men, is a novel finding. Evidence suggests that both burnout and empathy levels are higher in women, yet the authors of the present study did not note any ceiling effect in women’s empathy or burnout scores in the eligible studies, or greater variability in men’s empathy or burnout scores which could explain gender’s moderating effect. One possible explanation is that women’s empathy is more robust so that that they can maintain higher empathy even whilst experiencing higher burnout, but further research is warranted.

The non-significant findings of this meta-analysis are also of interest. Affective empathy was not statistically significantly related to any of the burnout sub-dimensions. The lack of association between affective empathy and emotional exhaustion provided the strongest support for the null hypothesis of any association analyzed in this meta-analysis, as shown by a Bayes Factor approaching zero. These results do not support the idea that higher affective empathy is associated with higher emotional exhaustion or lower emotional exhaustion. Affective empathy was primarily measured using the Empathic Concern component of the Interpersonal Reactivity Index, which assesses an individual’s ‘*feeling for*’ another individual [67]. Other questionnaires assessing affective empathy included the Toronto Empathy Questionnaire, and subscales of the Empathy Quotient, the Questionnaire of Cognitive and Affective Empathy, and the Basic Empathy Questionnaire. These questionnaires generally assess an individual’s ‘*feeling with*’ another individual [31]. The results related to affective empathy did not appear to depend on the questionnaire used or the conceptualization of affective empathy as ‘*feeling for’* or ‘*feeling with*’ another. However, the affective empathy questionnaires used in the included studies do not have the highest reliability and validity [31] and these associations had the highest heterogeneity scores. This provides some doubts in interpreting these results, and further research is warranted.

### Clinical implications

Although the results of this review do not provide evidence of direct causal links between empathy and burnout, they do indicate that in situations where medical student empathy is high, burnout is highly likely to be low, and vice versa. Given that medical student burnout is associated with poorer academic performance, increased rates of substance abuse, and impaired mental health [9], and higher medical student empathy is associated with higher personal accomplishment and clinical competence [68], medical educators are advised to create learning environments which foster empathy and reduce burnout. By creating preventative strategies to avoid burnout, maintaining the mental health of medical students, incorporating empathy-enhancing curriculums and promoting medical career sustainability, they can ensure quality of care for present and future patients.

### Recommendations for future research

The vast majority of studies present in the literature and available for analysis used self-reported empathy measures. Although these scales are helpful ways to measure internal empathic attitudes among medical students, patient-rated empathy scales can provide useful information on patients’ ability to detect these attitudes. Future studies could examine the associations between patient-rated empathy and burnout in medical students. It is also well-known that self-efficacy, i.e., the confidence in one’s ability to exert a certain behavior, is a reliable predictor of the actual behavior [69], and future studies could explore the possible associations between medical student empathy and their self-efficacy in exhibiting patient-centered behaviors in the interaction with patients [70].

The behavioral component of empathy was not analyzed due to a lack of studies measuring it (K = 1) but could provide useful information on the expression of medical student internal empathic attitudes. Future studies could examine the associations between behavioral empathy and burnout in medical students. It was also not possible to examine study year as a moderating variable as studies did not report results from single year groups. Given that empathy may decline as medical students progress through medical school and burnout may increase [7, 8], it may of use to observe the correlation between these two variables year-by-year.

### Study limitations

Some study limitations should be noted. First, the high *I*^2^ values suggest that a large proportion of the variation in results stems from underlying systematic differences between the available studies, rather than random error. While we identified two possible sources of the between-study variation, i.e., gender and response rates, the remaining included moderators either failed to explain a significant proportion of the variation or the data were not sufficient to conduct an analysis. Second, the included studies had assessed empathy and burnout with a range of different scales, diminishing across-study comparability and increasing between-study variability. Despite the fact that the most valid, standardized empathy measurement scales were used, the validity and reliability of these scales are still the topic of debate [31]. Thirdly, the majority of the studies included in this meta-analysis were cross-sectional in their study design and used bivariate correlations. Finally, burnout can be conceptualized in different ways, and it is possible that our merging of different concepts into one of the three MBI sub-dimensions: emotional exhaustion, depersonalization and personal accomplishment, may have reduced their explanatory power. For example, we combined the OBLI’s ‘disengagement’ sub-dimension with the MBI’s ‘depersonalization’. However, when rerunning the analysis separately for disengagement and depersonalization, it did not affect the results.

Due to only one study measuring behavioral empathy, we were not able to analyze its association with burnout. Further studies which categorize behaviors such as active listening and addressing patient emotions as behavioral empathy and analyze the association between these behaviors and burnout are required.

## Conclusion

Our study is the first systematic review and meta-analysis to examine the association between empathy and burnout in medical students. Our results confirm an overall negative relationship between empathy and burnout in medical students. Furthermore, cognitive empathy appears to be negatively associated with the burnout sub-dimensions of emotional exhaustion and depersonalization and most robustly positively associated with personal accomplishment. Affective empathy was not consistently associated with any of the burnout sub-dimensions. Future research should examine which modifiable parts of the medical learning environment could be altered to lower burnout and foster empathy.

### Supplementary Information

Supplementary Material 1. 

Supplementary Material 2. 

## Data Availability

Data is provided within the manuscript or supplementary information files. For extraction sheets, please contact the author Patrick Cairns patrickcairns@psy.au.dk

## Abbreviations

ESr: Effects size correlations
PROSPERO: The International Prospective Register of Systematic Reviews
MOOSE: The guidelines for Meta-Analysis Of Observational Studies in Epidemiology
PRISMA: Preferred Reporting Items for Systematic Reviews and Meta-Analyses
MeSH: Medical Subject Headings
PICO: Population, Intervention/Exposure, Comparator, Outcome
MBI: Maslach Burnout Inventory
BF: Bayes Factor
OSCE: Objective, structured, clinical examination
