# Supplementary figures and images for: The association between empathy and burnout in medical students: a systematic review and meta-analysis
Source: BMC Med Educ. 2024 Jun 7;24:640. doi: 10.1186/s12909-024-05625-6 (PMC11157786; doi:10.1186/s12909-024-05625-6)

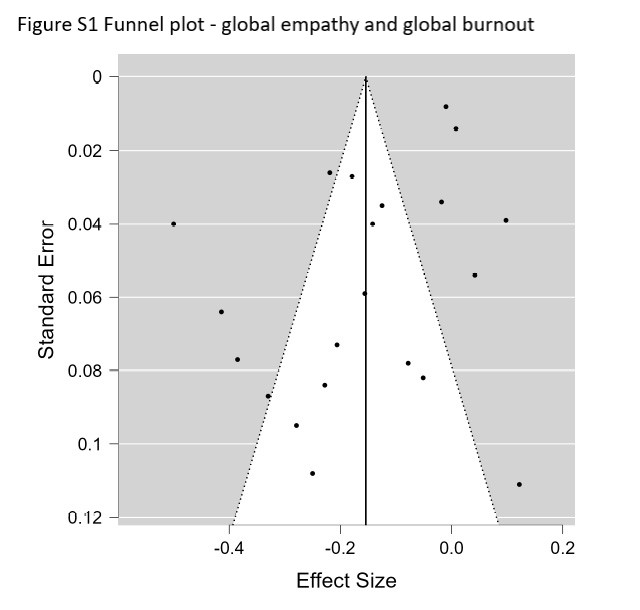

Supplement: Supplementary file 1 — Supplementary Material 1. [file 12909_2024_5625_MOESM1_ESM.jpg]
