# Supplementary material for: The association between empathy and burnout in medical students: a systematic review and meta-analysis
Source: BMC Med Educ. 2024 Jun 7;24:640. doi: 10.1186/s12909-024-05625-6 (PMC11157786; doi:10.1186/s12909-024-05625-6)
Supplement: Supplementary file 2 — Supplementary Material 2. [file 12909_2024_5625_MOESM2_ESM.pdf]

Supplementary Table S1 – Quality rating for included studies

Study (first author, year)	Item 1	Item 2	Item 3	Item 4	Item 5	Item 6	Item 7	Item 8	Item 9	Item 10	Item 11	Item 12	Item 13	Item 14	Quality Rating
Bigdeli et al. 2021	+	+	NR	+	+	-	-	+	+	NA	+	NA	NA	-	Fair
Bohler et al. 2021	+	+	NR	+	-	-	+	+	-	NA	+	NA	NA	-	Fair
Brazeau et al. 2010	+	+	+	+	-	-	-	+	+	NA	+	NA	NA	+	Fair
Cangussu Silva et al. 2018	+	+	+	+	-	-	-	+	+	NA	+	NA	NA	-	Fair
Capdevila-Gaudens et al. 2021	+	+	-	+	+	-	-	+	+	NA	+	NA	NA	+	Fair
Carrard et al. 2022	+	+	-	+	-	-	-	+	+	NA	+	NA	NA	-	Fair
Chae et al. 2017	+	+	+	NR	-	-	-	+	+	NA	+	NA	NA	-	Fair
Damiano et al. 2017	+	+	-	+	-	-	-	-	+	NA	+	NA	NA	-	Fair
DeWitt et al. 2016	+	+	-	+	-	-	-	+	+	NA	+	NA	NA	-	Fair
Dyrbye et al. 2021	+	+	-	+	-	+	+	+	+	+	+	NA	NA	+	Good
Gradiski et al. 2022	+	+	+	+	+	-	-	+	-	NA	+	NA	NA	-	Fair
Greenmyer et al. 2022	+	+	+	+	-	-	-	+	+	NA	+	NA	NA	-	Fair
Kilic et al. 2021	+	+	-	+	-	-	-	+	+	NA	-	NA	NA	+	Fair
Lucchetti et al. 2018	+	+	-	+	-	-	-	+	+	NA	+	NA	NA	-	Fair
Paro et al. 2014	+	+	+	+	+	-	-	+	+	NA	+	NA	NA	-	Fair
Shin et al. 2022	+	+	NR	+	-	-	-	+	+	NA	+	NA	NA	+	Fair
Stosic et al. 2022	+	+	-	+	+	-	+	+	+	NA	+	NA	NA	-	Fair
Suh et al. 2019	+	+	+	+	-	-	-	+	+	NA	+	NA	NA	-	Fair

von Harscher et al. 2018	+	+	+	+	-	+	+	+	+	+	+	NA	+	+	Good
Wercelens et al. 2023	+	+	+	+	-	-	-	+	+	NA	+	NA	NA	+	Fair
Wu et al. 2022	+	+	+	-	+	-	-	+	+	NA	-	NA	NA	-	Fair

Notes: NR = Not reported; NA = Not applicable; "+" indicates meeting criteria; "-" indicates not meeting criteria; Quality rating options, "Good", "Fair", or "Poor".

NIH Quality Assessment Tool for Observational Cohort and Cross-Sectional Studies

1. Was the research question or objective in this paper clearly stated?

2. Was the study population clearly specified and defined?

3. Was the participation rate of eligible persons at least 50%?

4. Were all the subjects selected or recruited from the same or similar populations (including the same time period)? Were inclusion and exclusion criteria for being in the study prespecified and applied uniformly to all participants?

5. Was a sample size justification, power description, or variance and effect estimates provided?

6. For the analyses in this paper, were the exposure(s) of interest measured prior to the outcome(s) being measured?

7. Was the timeframe sufficient so that one could reasonably expect to see an association between exposure and outcome if it existed?

8. For exposures that can vary in amount or level, did the study examine different levels of the exposure as related to the outcome (e.g., categories of exposure, or exposure measured as continuous variable)?

9. Were the exposure measures (independent variables) clearly defined, valid, reliable, and implemented consistently across all study participants?

10. Was the exposure(s) assessed more than once over time?

11. Were the outcome measures (dependent variables) clearly defined, valid, reliable, and implemented consistently across all study participants?

12. Were the outcome assessors blinded to the exposure status of participants?

13. Was loss to follow-up after baseline 20% or less?

14. Were key potential confounding variables measured and adjusted statistically for their impact on the relationship between exposure(s) and outcome(s)?

Reference:

National Institutes of Health (NIH). Quality Assessment Tool for Observational Cohort and Cross-Sectional Studies. National Heart, Lung, and Blood Institute. https://www.nhlbi.nih.gov/health-topics/study-quality-assessment-tools. Accessed January 12, 2024.
